# Prof. Joseph N. McDowell

**Published:** 1869-02

**Authors:** 


Prof. Joseph N. McDowell, M. D., late Professor of Surgery in the Missouri Medical College, St. Louis. This distinguished surgeon and medical teacher died Sept. 25th, 1868, having reached his 63d year. He was a man of marked ability, firm in his own opinions, untiring in his professional energy, and fearless of results. His death was sudden and unexpected, having died of congestive chill. May his name, his charities, and his many good deeds, live with a profession of which he while living was so bright an ornament.

